# Is the diagnostic radiological image an underutilised resource? Exploring the literature

**DOI:** 10.1186/s13244-019-0707-9

**Published:** 2019-02-06

**Authors:** William A. S. Cox, Penelope Cavenagh, Fernando Bello

**Affiliations:** 1 University of Portsmouth, James Watson West, 2 King Richard 1st Road, Portsmouth, PO1 2FR UK; 2 University of Suffolk, Ipswich, UK; 3 Imperial College London, London, UK

**Keywords:** Imaging, Diagnostic, Health benefits, Education, Patient, Communication, Behavior

## Abstract

The number of diagnostic imaging examinations being undertaken in the UK is rising. Due to the expensive nature of producing these examinations and the risks associated with exposing living tissue to the ionising radiation used by many of the imaging techniques, this growth comes with both a financial and a human cost.

In a time of limited resources, it is important that we are able to maximise the benefits which we extract from these resources. Therefore, a broad search of the current literature was undertaken to assess our current understanding of the nature of benefit available from diagnostic radiological images.

Two broad categories of benefit were identified: primary benefit (*n* = 470) and secondary benefit (*n* = 49). Primary benefits are those which are related to the justification for undertaking the imaging, e.g., abnormality detection, to assist in diagnosis or staging, or acting as an aid to clinical decision making, or intervention. Secondary benefits are those that are not related to the justification for imaging, e.g., to promote patient engagement and understanding or to facilitate communication.

Existing work considering primary benefits is comprehensive. Secondary benefit, however, is less well recognised and may not be reliably realised. Use of the image to realise these benefits has far-reaching potential. Particularly, there may be underexplored benefits which access to the images may provide to patients. This represents a gap in existing research which should be addressed.

## Key points

- The number of diagnostic radiological images being acquired is growing; this process involves both a financial and a human cost
- This work assesses existing understanding of benefits available from these images
- Primary benefits, e.g., diagnosis, intervention and guidance, are comprehensively understood
- Secondary benefits, e.g., communication facilitation, could be explored further

## Introduction

The number of diagnostic imaging examinations being undertaken in the UK is rising. 2016 alone saw growth of 2.1% [1]. Due to the expensive nature of producing these examinations [2] and the risks associated with exposing living tissue to the ionising radiation used by many of the imaging techniques, this growth comes with both a financial and a human cost.

Legislation dictates that each of the 40,654,715 examinations undertaken in 2016 was performed on the basis of a risk-benefit analysis [3]. Traditionally, expected benefits include the provision of abnormality detection, e.g., is there evidence of a fracture which will need treatment, or as an aid to clinical decision making, e.g., what type of fracture is present.

However, previous research has indicated that there are additional benefits available from these images [4]. Particularly, there may be underexplored benefits which access to the images may provide to patients.

It is important that we are able to maximise the benefits which we extract from these resources. According to benefits management theory, identification and structuring of benefits is the first stage in their realisation [5]. Moreover, it is important to establish whether there is a genuine imbalance in the literature with fewer studies pertaining to these additional benefits.

Thus, the questions for this review were:

1. What is the benefit of diagnostic radiological images?
2. To whom does the benefit accrue?

## Methodology

A structured narrative approach was chosen due to the potentially qualitative nature of the topic precluding the use of a systematic review [6]. This approach can promote reliability, trustworthiness and dependability, while minimising bias and error [6].

### Search strategy

Search terms were generated in alignment with the SPIDER (Sample, Phenomena of Interest, Design, Evaluation, Research type) tool. The SPIDER tool was selected as it was considered amongst a range of tools to be more effective in identifying qualitative and mixed methods studies. [7] This was important as this study was concerned with a range of benefits including those which are not medical but which are germane to this investigation.

This tool is designed to facilitate brainstorming of search terms and should ‘contribute to a more systematic process to qualitative evidence synthesis, improving researcher confidence that all relevant articles have been sought in the search process’ [8]. This review sought to investigate the potential benefits from imaging in the broad sense and was not primarily intended to address the potential benefits from specific modalities. The number of terms employed was restricted to those which arose through application of the SPIDER tool (Table 1) and were as below:

**Table 1:**
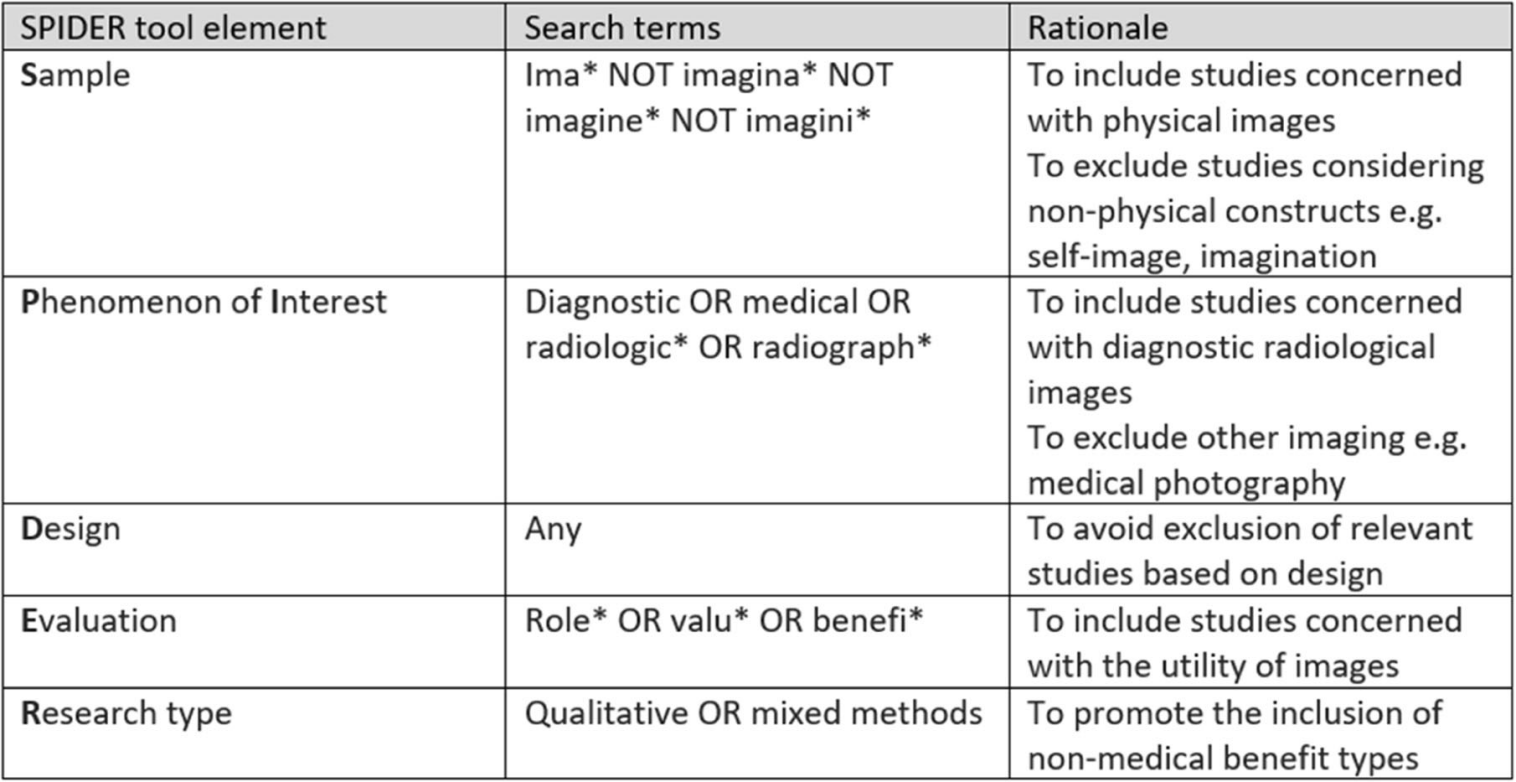
SPIDER search terms and rationale

### Data sources

In completing this review, the following databases were interrogated:

- CINAHL
- Cochrane library
- ProQuest
- PubMed
- Science Direct

### Screening

**Fig. 1:**
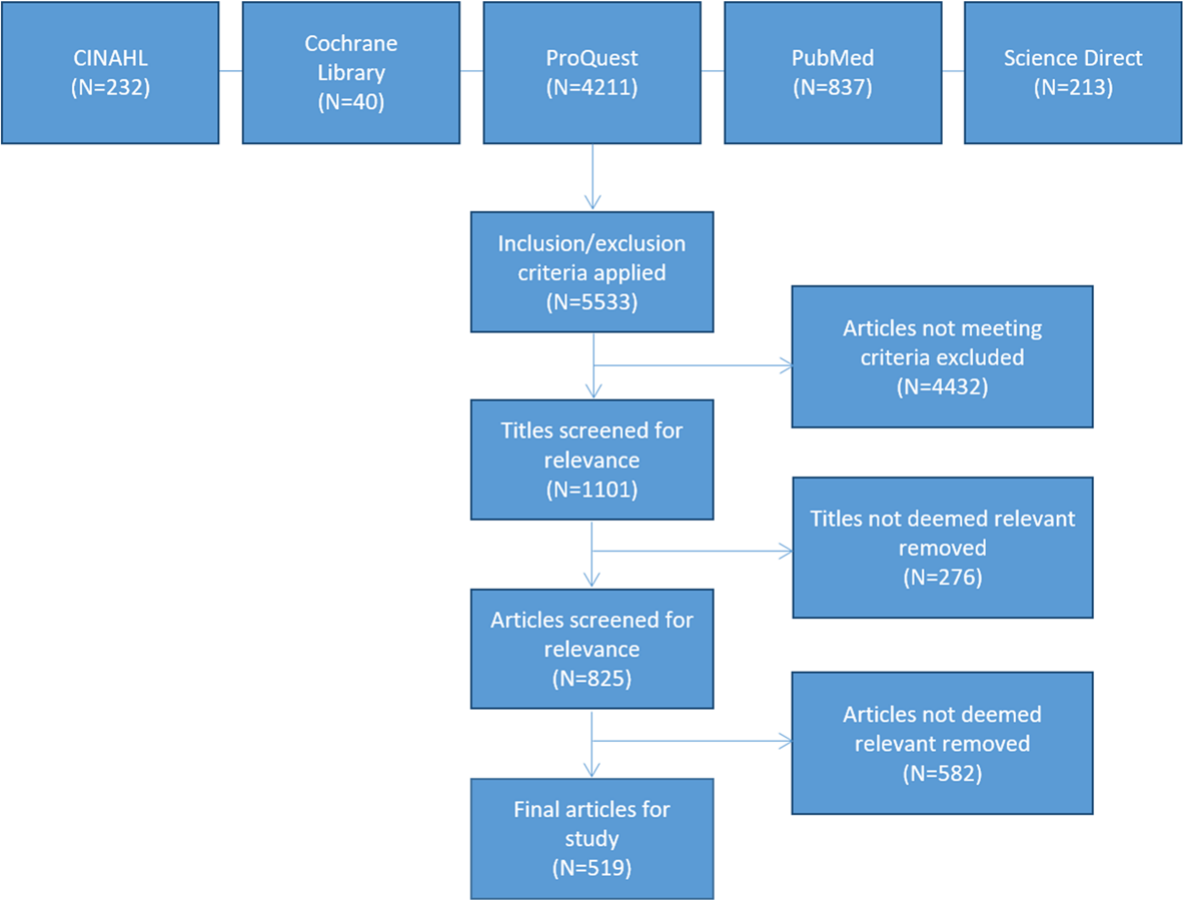
Adapted PRISMA diagram

**Fig. 2:**
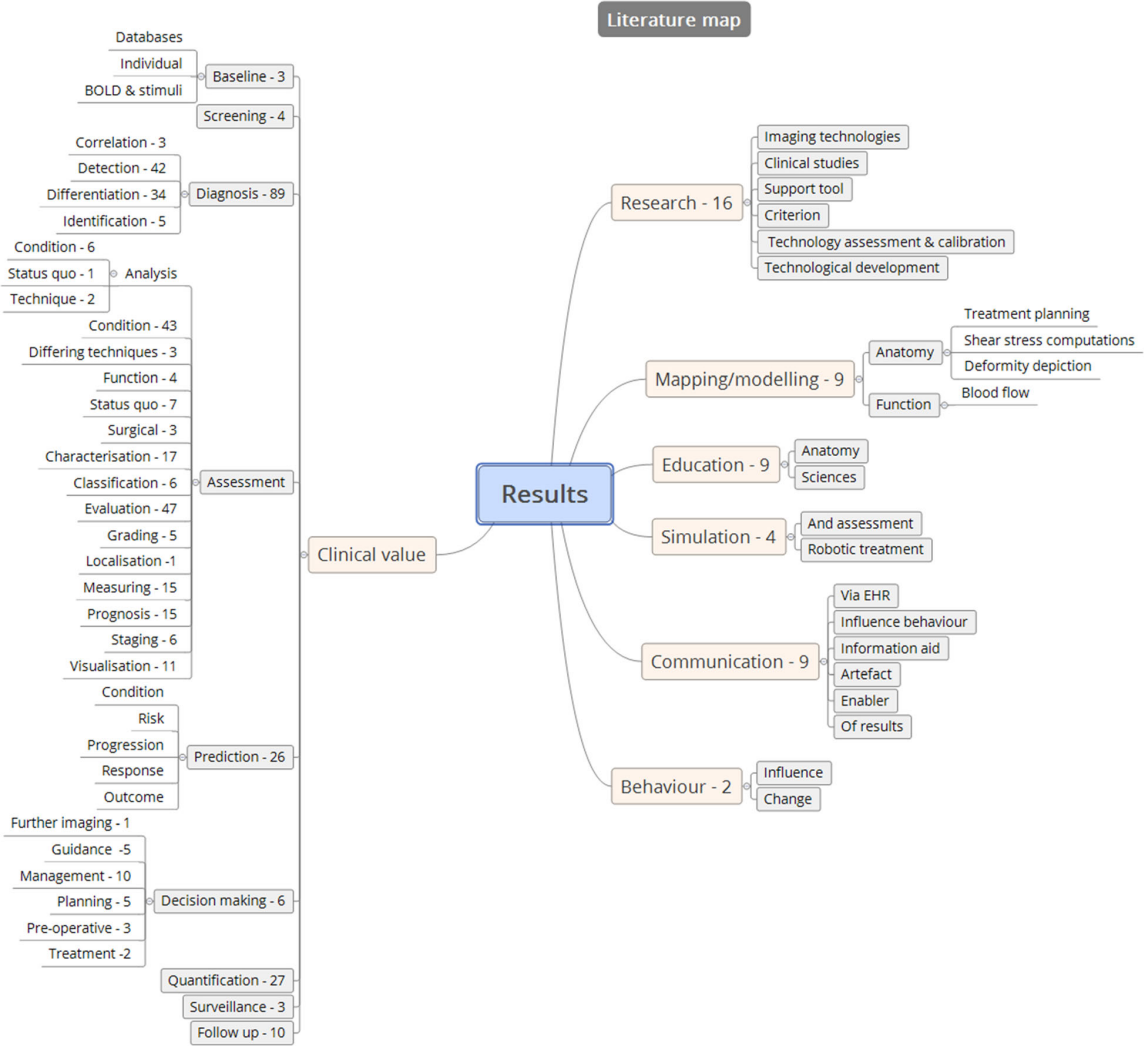
Literature map

Literature returned were screened for inclusion in alignment with the Preferred Reporting Items for Systematic Reviews and Meta-Analyses (PRISMA) guidelines [9] (Figs. 1 and 2). Although not a systematic review, the format provided by this validated instrument helped to shape the design of the review.

#### Inclusion criteria

Articles to be included were limited to those which concerned human imaging subjects, written in English and were published subject to peer-review within the last 10 years.

#### Exclusion criteria

Literature were excluded on the basis of relevance (concerned with metaphysical image constructs such as ‘self-image’) and scope (concerned with optical or cellular imaging).

## Results

A total of 5533 articles were returned. These articles were distributed across the databases as below:

- CINAHL—232
- Cochrane library—40
- ProQuest—4211
- PubMed—837
- Science Direct—213

Following application of the screening criteria, 519 articles were selected for analysis.

A meta-analysis of this data was deemed to be inappropriate as the explorative nature of the review entailed inclusion of diverse study designs, with differing outcome measures.

Articles were allocated themes extracted through familiarisation with the contents and based on the types of benefits described or addressed within the articles.

Following allocation of individual themes to the literature, two broad categories of benefit type were identified:

- Primary benefits (*n* = 470)
- Secondary benefits (*n* = 49)

### Primary benefits

The majority of literature returned (*n* = 470) was categorised as concerning primary benefits (see Table 2). Primary benefits are benefits extracted from the image which align with the rationale for its acquisition. Such benefits tend to fit with a traditional understanding of image value and may be further sub-categorised in alignment with the patient pathway temporally as follows:

- At the detection phase; for abnormality discovery
- At the diagnostic phase; for condition identification and further assessment
- At the management phase; for decision making, intervention or follow-up

**Table 2:**
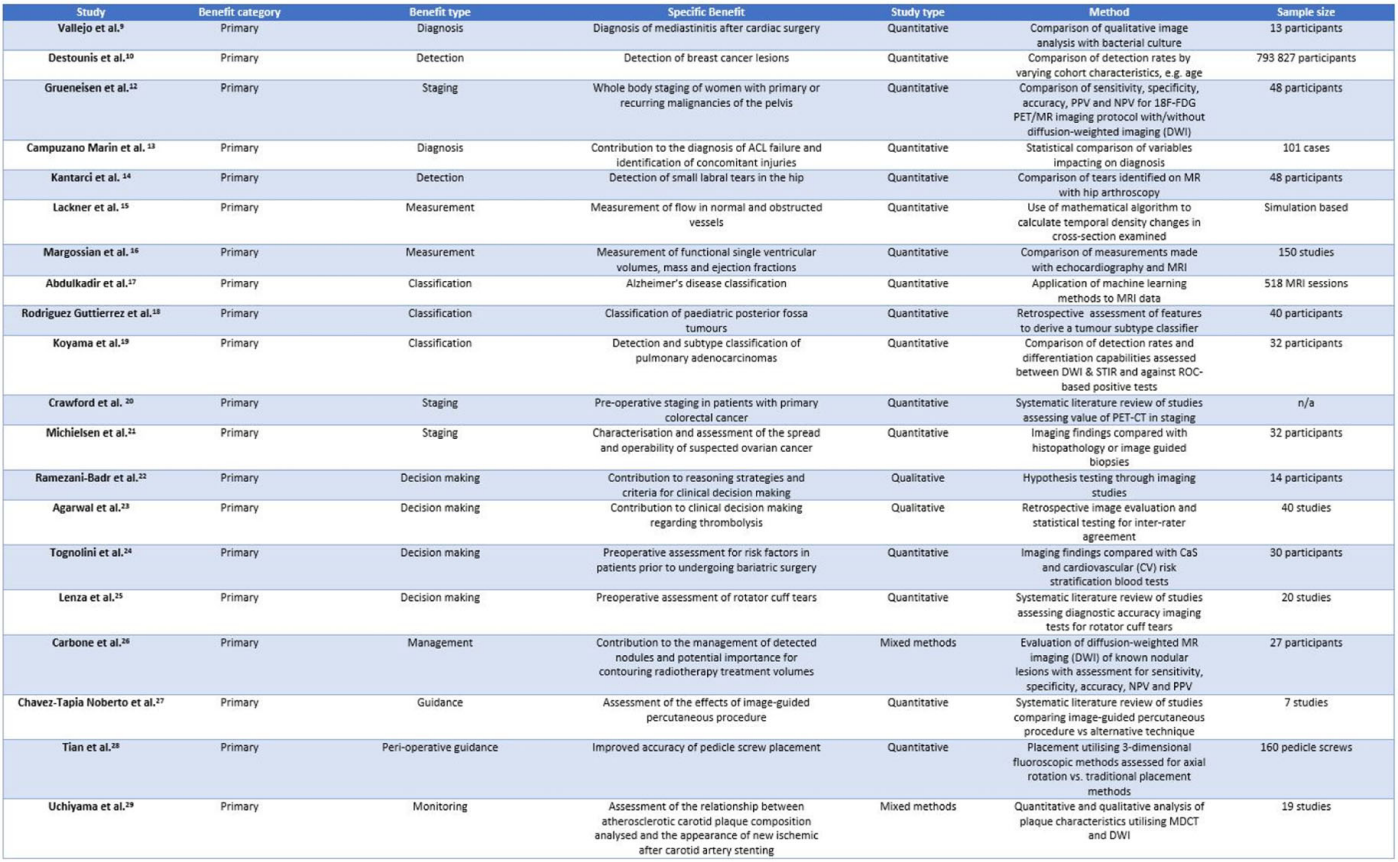
An overview of study types and contents and the primary benefit categories

An in-depth analysis of all of the facets of clinical benefit in images is beyond the scope of this article. However, the types of benefits which contributed to each phase are briefly described below:

#### Detection phase

This phase includes a range of benefits covering not only detection of abnormalities, for example, the clinical utility of 99mTc-labelled ubiquicidin 29–41 antimicrobial peptide for detecting mediastinitis following cardiac surgery [10] or the assessment of accuracy of dual-time-point 18F-FDG PET, but, further, the contribution of imaging to the ruling out of abnormalities, as well as its use as a screening tool in both public health initiatives such as the breast screening programme [11], and private health M.O.T. packages [12].

#### Diagnostic phase

This phase deals with benefits which the image may contribute once the presence of an abnormality has been confirmed. Such benefits include the contribution of the image to the formulation of a diagnosis [13, 14], the measurement [15–17] or classification [18–20] of the abnormality and the contribution of imaging information to disease staging [21–23].

#### Management phase

This phase is concerned with how the patient is subsequently managed. Images have a recognised role in supporting interventional procedures and other healthcare-related activities either pre-, peri-, or post-intervention. Pre-interventional benefits include contribution to surgical planning [24, 25] and decision making [26, 27], peri-interventional benefits are those concerned with image guided procedures such as biopsies or surgery [28, 29], and post-interventional benefits include the contribution of images to follow-up and monitoring processes [30, 31].

### Secondary benefits

Beyond the primary benefit types described above, there are various secondary benefit types recognised within the literature (see Table 3). These are the types of benefit which are not necessarily related to the purposes for which the images were originally intended. This benefit is less well recognised and often less tangible. The literature describes these forms of benefit infrequently and seldom directly. They may be categorised as:

- Educational benefit
- Relational benefit
- Technological benefit

**Table 3:**
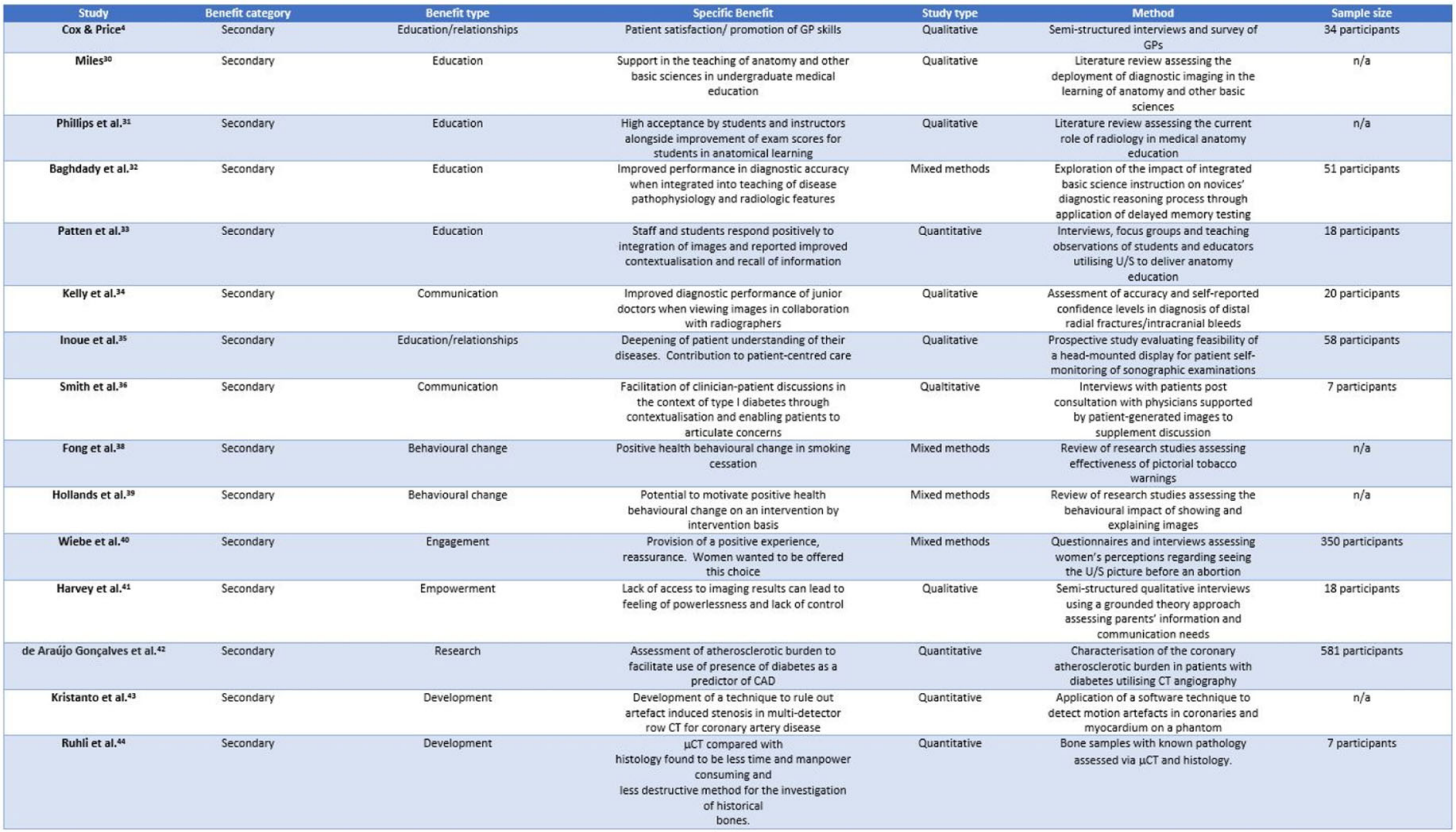
An overview of study types and contents and the secondary benefit categories

#### Educational benefit

Images have traditionally been used in the teaching of anatomy to health students. However, the breadth of areas where images can contribute benefit in the educational sense is gradually increasing [32]. This expansion of understanding has implications for our thinking around the benefit which images may contribute. For example, the benefit which radiological images contribute to the teaching of anatomy and physiological processes is recognised within the literature [33]. The use of images in this sense promotes understanding through providing context [34]. Beyond their benefit in this reference oriented sense, images, or the production of images, can be used directly in anatomical instruction. Ultrasound (U/S), for example, when used as a tool for teaching abdominal anatomy, was valued by students as a means for reinforcing their existing academic knowledge through hands-on clinical contextualisation [35]. Further, the growing availability of 3D imaging datasets allows students to visualise time series imaging studies displaying motion of either organs, e.g., the beating heart [32], or substances. Thus, images can be used to demonstrate both structural and process-based/functional information in both health and disease. Baghdady et al. [34] found that incorporation of the image in this sense had a significant effect on learning (*p* = 0.01). Groups who were taught basic science with links between disease pathophysiology and radiologic features integrated with imaging outperformed groups who were delivered segregated basic sciences in diagnostic accuracy. While this is a single study, and there is a need for further work, this does support the suggestion that a role exists for radiological images in teaching in this area.

#### Relational benefit

Building on and extending educational benefit is relational benefit. Relational benefit in this context refers to the potential for images to contribute to the relationships between stakeholders. The contribution of relational benefit by diagnostic images may be categorised as promoting the following:

- Communication
- Engagement

##### Communication

The image has been indirectly recognised for its benefit as an artefact of communication, providing a focal point for discussion both between clinicians [36] and between clinicians and their patients [37]. Shared interpretation of radiographs by clinicians in the accident and emergency (A&E) environment resulted in area under the curve (AUC) scores for interpretation improving significantly for both conventional and CT images [36]. Additionally, GPs perceive images as useful for communicating with, and providing reassurance to, patients [35]. Images have also been shown to facilitate clinician-patient discussion regarding patients’ condition [38] and have the potential to allow patients to articulate concerns and ask questions. This has been argued to result in decreased patient anxiety, increased patient confidence in services, and promoting the development of a partnership-based approach to their care [37].

##### Engagement

Engagement is another avenue via which relational benefit may be demonstrated. Engagement in this context may be defined as the contribution that diagnostic radiological images make to the enablement of stakeholders in the processes surrounding the healthcare encounter. These benefits may accrue to any stakeholder, but are particularly pertinent to patients and other non-clinical stakeholders. This engagement may be evidenced through the promotion of behavioural change, patient empowerment and satisfaction.

The role of visual stimuli in influencing human behaviour has been widely explored in several academic fields, including marketing and preventative medicine [39]. A pertinent example is the significant impact on smoking cessation realised through use of vivid imagery on cigarette packages [40]. A Cochrane review from 2010 directly assessed the impact of visual feedback of individuals’ medical imaging results on changing their health behaviours. The review included two trials utilising U/S images and two trials utilising CT images. The trials assessed whether the use of images in the feedback of test results could influence subject behaviour against various outcomes [41]. While this review does demonstrate some recognition of the potential for images to promote behavioural change, the findings were not conclusive. The authors noted that the volume of available evidence was limited and further work is required in this area [41].

Beyond behavioural change, images promote the empowerment and satisfaction of non-clinical stakeholders. In one study, women volunteered a desire to see pre-abortion U/S images of their foetuses [42]. While this access did not measurably influence the decision to undergo the procedure, the women did note that access to the images helped them to feel empowered and in control of the situation [42]. Empowerment was also cited as a factor in the importance of access to images by parents of children in the neonatal unit (NNU) in a study assessing levels of information and communication provision from care providers regarding their children [43]. Patients report that, on occasion, they do not feel informed about the results of diagnostic tests including diagnostic imaging leading to feelings of powerlessness and lack of control [43].

#### Technological benefit

Finally, the literature describes benefit which may be extracted from images in a technological sense. Beyond a well-developed tradition of using imaging in clinical research, for example, utilising images as a predictor of pathology [44]; contributing to the development, refinement and calibration of existing technologies [45]; or providing a basis upon which to compare and refine existing techniques [46], images may contribute to the development of novel technologies such as the production of models of organs through 3d printing based on data acquired from diagnostic radiological imaging procedures. Recently, companies including M3dia Studio [47] and 3dprinting.com [48] have been producing such models. These in turn may be utilised to inform new or improved imaging techniques in order to answer specific questions [47, 48]. Techniques such as 3d reconstruction have previously played a role in surgical planning, but, if internal architecture can be copied and reproduced through 3D printing, the benefit of imaging for surgeons to practice in specific cases is all the more powerful.

## Discussion

The majority of the literature returned was concerned with primary benefit forms (*n* = 470 of *n* = 519). This is overwhelmingly clinical in nature and was recognised as being comprised of tangible benefits, which are clinically orientated and tend to be measurable. These benefits tend to accrue directly to clinicians.

Secondary benefit was less well developed. This was defined as being comprised of benefits which were not intended for extraction at the point of image acquisition and tended to be comprised of less tangible benefits, which are difficult to measure. Secondary benefits, however, offer several novel opportunities under three broad benefit subtypes: educational benefit, where images were noted as having a positive impact on recall and understanding; relational benefit, where images were recorded as promoting education and engagement; and technological benefit, where images contributed to the development of both novel technologies and techniques.

Technological benefit is likely to be an area of growing importance with developments in Artificial Intelligence (AI) having the potential to impact on radiology in a number of key areas including automated detection. Many of these capabilities benefit from the increasing availability of datasets to improve their reliability and, therefore, their clinical utility both in improving the diagnostic value of the images themselves and in potentially contributing to alleviating pressures rooted in workforce shortages.

Additionally, secondary benefits may accrue directly to a wider stakeholder group. There is little consideration within the existing literature of how patients, for example, may directly access benefits from their own images. Indeed, no work directly asks what stakeholders actually perceive as the benefit of diagnostic radiological images. This may be as a function of stakeholders lacking avenues for accessing such benefit. However, access to patient portals is increasing and, therefore, an understanding of stakeholder perceptions of potential benefit from diagnostic radiological images, particularly pertaining to stakeholders external to the clinical environment, is becoming increasingly pertinent. Furthermore, there are important questions to be answered surrounding how such access might be managed in order to enable stakeholders to realise potential benefits while mitigating any inherent risks.

### Limitations

While the search terms and data sources for this review were deliberately broad, there remains a risk that relevant articles may have been missed. While this review exclusively utilised the SPIDER tool in identifying literature, it is possible that using additional search tools may have increased the number of articles identified. Furthermore, no literature explicitly asks the question of what benefits are available from diagnostic radiological images. As such, the benefits listed were identified through a categorisation process which may reflect the bias of the reviewers.

In considering secondary benefits, it is important to note that, while potentially important, there should be no confusion as to the role these benefits play in the justification of imaging. Secondary benefits can only be considered as being supplementary in nature and imaging should not be undertaken based on the potential to realise these benefits.

## Conclusion

The existing work which addresses primary benefit in images is comprehensive. However, beyond the primary benefit of images, there are a number of secondary, or recycled, benefits available. For example, the literature indicates the potential to use the diagnostic radiological image to promote education or to enhance and promote communication and engagement. Such usage has wide ranging potential benefits. The use of the image as an artefact for interpersonal communication, for example, may prospectively act as an information aide or adjunct, assist with conveying findings, provide reassurance and help to deliver personalised care. Additionally, there is some evidence that diagnostic radiological images have benefit as a tool to influence health behaviour. Furthermore, a Cochrane review conducted on this subject explicitly calls for further work in this area [41].

Despite the above, however, the potential of the image for realising a secondary benefit remains largely underexplored, an incidental by-product of the imaging process. This is a gap which should be addressed through further research.

## Abbreviations

A&E: Accident and emergency
AUC: Area under the curve
NNU: Neonatal unit
PRISMA: Preferred Reporting Items for Systematic Reviews and Meta-Analyses
SPIDER: Sample, Phenomena of Interest, Design, Evaluation
U/S: Ultrasound
